# Internal Migration and Loneliness in Childhood: The Moderating Role of Family Structure and Cultural Individualism

**DOI:** 10.1007/s10680-025-09741-x

**Published:** 2025-07-21

**Authors:** Aude Bernard, Patricia McMullin, Sergi Vidal

**Affiliations:** 1 Queensland Centre for Population Research, The University of Queensland, St Lucia, Australia; 2 INVEST Flagship Research Centre, University of Turku, Turku, Finland; 3 Centre for Demographic Studies CED-CERCA & Department of Sociology, Autonomous University of Barcelona, Barcelona, Spain

**Keywords:** Internal migration, Residential mobility, Social networks, Europe, Lifetime migration, Survey of Health, Ageing and retirement in Europe

## Abstract

The negative impact of childhood internal migration on diverse life outcomes is well documented. The main hypothesis to explain this association is the severance of social ties. However, empirical evidence on the link between internal migration and loneliness in childhood is critically lacking. We address this gap by establishing the association between childhood loneliness and inter and intra-regional migration in Europe. Using retrospective life-history data from the Survey of Health, Ageing and Retirement in Europe in 26 countries, we run a series of country-fixed effect logistic regression for cohorts born before 1967. We find that the probability of often being lonely is 25–39 per cent higher among children who moved at least once, although having siblings exert a strong protective effect. The strength of the association between loneliness and internal migration dissipates with duration of residence, suggesting that children progressively adjust to new surroundings, particularly if they migrated before starting school. However, the mitigating role of duration of residence diminishes with the number of past moves. Despite the limitations of retrospective survey data, our results show a clear association between internal migration and loneliness, highlight the increased risks of chronic migration among children and lend support to the loss-of-social-networks theory, particularly in individualist societies where friendship plays a greater role in social networks and where children consistently report higher levels of loneliness.

## Introduction

The negative consequences of childhood internal migration are well documented. They include lower mental health (Rumbold et al., 2012; Tseliou et al., 2016), poorer school performance (Mollborn et al., 2018; Ziol‐Guest & McKenna, 2014) and lower educational attainment (McMullin et al., 2021; Metzger et al., 2015). Some of these effects are felt into adolescence and early adulthood (Myers, 1999a; Tønnessen et al., 2016; Tseliou et al., 2016; Webb et al., 2016), including a higher risk of substance abuse (Böhlmark, 2009) and depression (Tseliou et al., 2016).

The strength of these associations is, however, moderated by the timing of internal migration (Fowler et al., 2015; Myers, 1999a; Rumbold et al., 2012), the distance moved (Swanson & Schneider, 1999) and the number of moves (Pribesh & Downey, 1999; Tseliou et al., 2016; Tucker et al., 1998), suggesting that it may be the cumulative effect of repeated moves over a sustained period of time that results in poorer life outcomes. More importantly, some of these effects dissipate when family arrangements and parental circumstances are considered (Anderson et al., 2014; Gambaro et al., 2022). This suggests that it might not be migration itself that is detrimental to children, but the causes underpinning it such as parental separation, periods of unemployment and housing instability (Gambaro & Joshi, 2016; McMullin et al., 2021). Thus, some studies have found no association, while others have shown a positive relationship, including long-term educational benefits, particularly when residential mobility is associated with a change of school (Hango, 2006; Swanson & Schneider, 1999; Vidal & Baxter, 2018).

While evidence remains mixed, three main mechanisms have been proposed to explain why moving as a child might be harmful. Grounded in strain theory, the first line of explanation emphasises the strain caused by migration, which is a stressful event in its own right (Haveman et al., 1991). Short-term stress in childhood can result in a disjunction between expectations and achievements that can lead to deviant behaviour (Agnew, 1992) when children lash out at parents who are blamed for the move. This mechanism is supported by evidence of poorer mental health (Rumbold et al., 2012; Tseliou et al., 2016) and behavioural issues (Böhlmark, 2009; Simpson & Fowler, 1994) following internal migration.

The second and most common explanation is the loss of social networks—both family and school friends and neighbours—caused by internal migration. The disruption of important relationships, particularly if they are positive role models, can in turn lead to poorer life outcomes. Indeed, the role of social capital in educational achievement and attainment is well documented and extends to both family and community social capital (Israel et al., 2001) and friends (Crosnoe et al., 2003). The third type of explanation—closely related to the second—draws on acculturation theory (Berry, 1997), which emphasises the cultural and psychological costs of adapting to a new environment. This theoretical lens is often used to study childhood loneliness among emigrants (Leondari, 2001) and rural–urban migrants in countries like China (Kuo et al., 2021). Empirical research shows that the loss of social networks increases the risk of perceived loneliness among international migrants compared to host populations. Key mechanisms underlying these feelings of loneliness include the lack of new social support networks, the structure and characteristics of existing networks, the frequency of contact and overall satisfaction with social relationships (Fokkema & Naderi, 2013; Ten Kate et al., 2020; van Tilburg & Fokkema, 2021). Similar associations have been found for migrant groups that share characteristics with internal migrants, such as intra-European migrants who do not face legal or work-related restrictions and migrants with a local partner (Koelet & de Valk, 2016). Research on internal migration in China further supports these findings, demonstrating that the disruption of established social networks significantly contributes to loneliness. For instance, studies on rural-to-urban migrant children show that weakened social support systems are a major factor influencing their sense of social isolation at destination (Chen & Yang, 2022).

Thus, we expect loneliness to be positively associated with migration (H1a), but this association to diminish with duration of residence as children adjust to their new surroundings and build new social networks (H1b). For this reason, we expect only migration during school years—as opposed to earlier in life—to be linked to greater loneliness (H1c). We also anticipate loneliness to increase with the number of moves because of the repeated severance of social ties (H1d) and long-distance moves to be more strongly associated with loneliness than short-distance relocations (H1e), which are less likely to affect social networks.

Because family members are effective at providing social support (Taniguchi & Kaufman, 2022), we also anticipate family structure to exert a protective effect on migration-induced loneliness. In particular, we expect post-migration loneliness to be lower in multi-child families (H2a) as siblings can support one another (Ponzetti & James, 1997) and in two-parent families (H2b) because of the availability of both parents to support their child after moving (Pasteels & Bastaits, 2020) and because increased loneliness following parental separation (Steinbach & Augustijn, 2023), which may compound migration-induced loneliness.

Importantly, there is evidence of cross-national variation in the prevalence of loneliness—both in the general population and among children and adolescents—depending on the level of cultural individualism versus collectivism (Barreto et al., 2021). Countries with cultural orientations towards individualism in the North and West of Europe value self-reliance and encourage loose social networks dominated by chosen relationships such as friends. In contrast, collectivist cultures in the South of Europe support tighter social networks dominated by family members (Hofstede, 1998). Some studies have found lower level of loneliness in individualist countries compared with collectivist countries (Dykstra, 2009) because of higher social needs in collectivistic societies can affect the match between expected and realised social relationships (Johnson & Mullins, 1987). Other studies have found higher loneliness in individualist countries, particularly among young adults (Barreto et al., 2021). In the context of childhood migration, we anticipate a higher prevalence of loneliness in countries with cultural orientations towards individualism because internal migration predominately affects friendship ties which are more central in individualist cultures (H3a). We also anticipate the impact of migration on loneliness to be comparatively lower in collectivist countries because of the greater role of family relationships and support (H3b), which can to some extent compensate for the loss of friendship ties (Lyell et al., 2020).

Despite the ubiquity of the social-network-loss type of explanation, it remains difficult to empirical quantify the mediating role of social networks on loneliness following an internal move. Very few longitudinal studies collect detailed information on the social networks of children. Instead, there are small-scale ad hoc surveys often focussed on selected regions within countries (Kuo et al., 2021), which makes it difficult to generalise findings. We know, however, that internal migration in adulthood is associated with a higher level of loneliness (Oishi et al., 2012) and smaller social networks centred on friends (Drevon et al., 2021). Endogeneity and reversed causality are key challenges when seeking to understand the association between loneliness and internal migration among adults. Social psychologists circumvent this issue through experimental studies by asking people to anticipate the consequences of mobile lifestyle (Oishi et al., 2013). In this paper, we take a different strategy and instead focus on children who are tied migrants and have limited say in their migration experiences. They do not enact a preference they already hold when migrating, but they are rather exposed to migration although parents may make residential choices based on their children’s response to a potential move. Another reason to focus on childhood migration is the long-lasting consequences of childhood loneliness, which is a strong predictor of depressive symptoms in adolescence (Qualter et al., 2010) and poor health in young adulthood (Caspi et al., 2006), and whose root causes warrant a deeper understanding.

In that context, we leverage retrospective life-history data collected in 2017 in 26 European countries as part of Wave 7 of the Survey of Health Ageing and Retirement in Europe (SHARE) to establish the association between loneliness and internal migration in childhood and to determine the moderating role of family structures and cultural individualism. Respondents were born before 1967 and aged 50 years and over when asked to report their complete migration history since birth along with the educational, employment, marital and parental history. Respondents were also asked to report their childhood circumstances, including feelings of loneliness, living arrangements and socio-economic status. Thanks to its cross-national structure, this dataset has the unique advantage of allowing us to estimate the association between internal migration and loneliness while taking into account the role of cultural orientations towards individualism and collectivism. Yet, it faces several limitations, including the self-reported nature of events and feelings that occurred decades earlier, which face the risk of recall bias and post hoc rationalisation which we discuss, among other challenges, in the Data and Methods section and go back to in the Conclusion.

Using regression analysis, we establish the association between internal migration history between birth and age 16 and self-reported loneliness during school years, while controlling for a range of socio-economic characteristics. We do so for both intra- and inter-regional migration defined as moves between and within NUTS-2 regions, which is the second tier of the Nomenclature for Territorial Units for Statistics and allows us to distinguish between short- and long-distance migration. We also distinguish between pre-school moves and moves that occur during school years and we use a series of interaction terms between number of childhood moves and (a) duration of residence, (b) having siblings, (c) presence of both biological parents, and (d) cultural individualism to test our research hypotheses. We conduct sensitivity analysis to ensure that the results are robust to (a) the choice of control variables, (b) the self-reported nature of our measure of loneliness and (c) model specifications. More information on the dataset and our analytical strategy is provided in the next section. After presenting the results in Sect. 3, we conclude by discussing the cumulative effect of multiple moves on loneliness, the attenuating role of longer durations of residence and the moderating role of family structure and cultural individualism. We end the paper with thoughts on the implications of our results for migrating children and suggest directions for future research.

## Data and Methods

### Survey of Health, Ageing and Retirement in Europe

To establish the association between internal migration loneliness in childhood, we draw on retrospective life-history data from the Survey of Health, Ageing and Retirement in Europe (SHARE), a compendium of nationally representative surveys of the adult population aged 50 and older in Europe (Börsch-Supan, 2020b). The data are publicly available to registered users at www.share-eric.edu. In 2007–08 (Wave 3), SHARE retrospectively collected information on the complete life histories—including residential histories—of respondents from 11 countries. In 2017 (Wave 7), this retrospective data-collection process was repeated for new survey participants—that is individuals from the 14 countries that joined SHARE after 2008 and individuals from refreshment samples. Thus, SHARE has collected information on each participant’s life-history maximum once. The childhood circumstances module was extended in Wave 7 to elicit information about the respondent’s experience of loneliness. We therefore restrict the analysis to Wave 7 data, which was collected in 26 countries, namely Austria, Belgium, Bulgaria, Croatia, Cyprus, the Czech Republic, Denmark, Estonia, Finland, France, Germany, Greece, Hungary, Italy, Latvia, Lithuania, Luxembourg, Malta, Poland, Portugal, Romania, Slovakia, Slovenia, Spain, Sweden and Switzerland. To avoid left censoring bias and ensure that all respondents were equally exposed to the risk of migrating internally within the survey country in childhood, we exclude foreign-born individuals. We are left with an analytical sample of *n* = 37,042.

The retrospective nature of the survey and its focus on events that took place decades earlier may lead to poor recall (Looker, 1989). For this reason, education, employment, partnership, fertility and residential histories were collected using life-history calendar (LHC) grids (Axinn et al., 1999; Blane, 1996; Brüderl et al., 2017). This technique involves collecting responses using a schematic form, where columns represent successive years of life since birth alongside national and international historical events, which act as temporal landmarks to facilitate recall (Axinn et al., 1999; Blane, 1996; Brüderl et al., 2017). Rows represent the different life domains against which retrospective data are collected. This sequential, cross-dimensional visual representation of the life course has the advantage of displaying both events (e.g. migration) and spells (e.g. duration of residence), which reduces recall errors associated with forward and backward telescoping (Gaskell et al., 2000; Glasner et al., 2012), time expansion and event omission (Glasner & van der Vaart, 2008). The accuracy of this data-collection approach is supported by population-level incidence of life-course transitions obtained from retrospective survey data comparable to that sourced from longitudinal survey (Börsch-Supan, 2020a). These include salient childhood events in the distant past, such as migration (Smith, 2009).

Migration, which is the focal control variable, is defined as a change of residence within NUTS-2 region (intra-regional migration) and a change of NUTS-2 region (inter-regional migration). SHARE collected up to 30 changes of address for durations of residence of at least 6 month since birth, recoding NUTS-2 region of residence for each change of address. Panel A of Table 1 reports the number of internal migrations from birth to age 16. It shows that internal migration is a rare event. Close to 7 in 10 respondents did not change address within NUTS-2 regions and over 9 in 10 did not change NUTS-2 region of residence. Among childhood migrants, the majority migrated once although a small proportion migrated multiple times. Over 10 per cent moved within NUTS-2 region at least twice. Panel B of Table 1 compares the level of migration before age 6 and during school years. In line with the age schedule of migration, a higher proportion of respondents moved during school years than early childhood when internal migration and residential mobility are low (Rogers & Castro, 1981). These results also align with estimates of childhood mobility from SHARE (Bernard & Vidal, 2020; Pertzikovitz et al., 2024).

**Table 1:** Childhood migration history

	Intra-regional migration			Inter-regional migration		
Number of moves	N unweighted	% unweighted	% weighted	N unweighted	% unweighted	% weighted
*PANEL A: Number of moves from birth to age 16*						
0	28,181	76.05	73.81	33,646	90.79	91.31
1	6,272	16.92	18.25	2,741	7.40	9.64
2	1,854	5.00	5.67	526	1.42	1.11
3	5,01	1.35	1.47	145	0.39	0.30
4	250	0.67	0.81	–	–	–
*PANEL B: Timing of migration*						
Moved at least once before age 6	3,428	9.25	10.09	1,247	3.36	3.51
Moved at least once after age 6	6,920	18.67	20.47	2,503	6.75	6.05

*Source*: Authors’ calculations from wave 7 of SHARE

As part of the retrospective module, respondents were also asked how often they were lonely for friends during their school years (up to 16 years of age) on a four-point scale—often, sometimes, rarely and never. This question was derived from a question from the Panel Study of Income Dynamics (PSID) Retrospective Circumstances Study in 2014, which has been used to study the determinants and consequences of childhood loneliness in the United States (Heidinger & Willson, 2019). We collapse these responses into a binary variable—0 not often lonely and 1 often lonely to simplify results reporting. As much as 6.64 per cent of the sample (of 5.66 unweighted) reported being often lonely in childhood. As a robustness check, we run additional regression analysis with the original four-point measure for loneliness using ordinary least square regression, ordered logit and multinomial regression in Appendices A and B.

Our measure of childhood loneliness may be biased because of post hoc rationalisation, which often affects the recall of past events and circumstances, including adverse childhood experiences (Hardt & Rutter, 2004), and may result in earlier experiences of loneliness be re-interpreted based on current experiences of loneliness. In other words, old loners may be more likely to report being lonely in childhood whereas old socialites may be less likely to report early-life loneliness because they assess their past situation based on their present circumstances. Thus, as a robustness check, we replicate the analysis separately for two sub-groups: those who reported feeling often lonely at the time of the survey and those who did not. Results in Appendix C show that the association between loneliness and internal migration is statistically significant for both groups, which indicates that our results are not significantly affected by the retrospective nature of childhood loneliness. Another limitation relates to the fact that the timing of each migration was collected, but the question on loneliness refers broadly to school years. Thus, we do not know if loneliness increased before or after a move, how long the feeling of loneliness lasts, or its intensity. That is why we refrain from making any claims about causality and go back to this limitation in the conclusion.

### Modelling Strategy

In our baseline model, Model 1, we define migration as having moved at least once either within or between NUTS-2 from birth to age 16. In Model 2, we replace our binary measure of migration by the number of migrations to determine whether the repeated severance of social ties is detrimental to children and is associated with higher loneliness. Next, in Model 3 we replace migration history by duration of residence at age 16 to establish whether the impact of migration on loneliness abates as children progressively adjust to their surroundings and develop new friendships. We then interact the number of migrations with duration of residence to gauge whether the impact of past migration events persists as duration of residence increases. In Model 4, we explore the role of migration timing by distinguishing between pre-school moves before age 6 and moves during school years. Because early movers are more likely to move repeatedly during childhood (Bernard, 2022; Myers, 1999b), we focus on one-time movers and compare them to children who never moved.

In the last set of analysis, we explore whether family structure and cultural individualism act as moderators. We do so in Model 5 by adding interaction terms between our binary measure of migration and the presence of (a) both biological parents and (b) siblings. In Model 6, we add an interaction term between our binary measure of migration and cultural individualism using Hofstede (1984)’s measure of individualism versus collectivism.

We estimate the association between loneliness and internal migration in a regression framework. Our logistic regression model includes a range of control time-fixed variables: sex, birth cohort (before, during and after WWII), having foreign-born parents and survey country. The latter allow us to control for fixed structural conditions such as kinship systems that may affect loneliness among children. We elected a country-fixed effect instead of a multi-level framework because the number of countries is below 30, which can lead to unreliable country effects (Bryan & Jenkins, 2013; Möhring, 2012). Furthermore, we do not replicate the analysis for each country separately because of the small size of national samples, which carry the risk of Type II error. In Model 6, we replace the country-fixed effect by Hofstede (1984)’s measure cultural individualism, which is now available for 80 countries around the globe (Hofstede, 2001) and has the advantage of being relatively stable over time in so far that countries’ scores relative to other countries have not changed over time (Beugelsdijk et al., 2015). It is a continuous measure that ranges globally from a high of 94 in the USA to a low of 6 in Guatemala. Within our sample, individualism varies from a high of 76 in Italy and 75 in Denmark and Belgium and a low of 27 in Portugal and Slovenia followed by 33 in Croatia and 35 in Greece. Scores are obtained from responses to a series of standardised questions from nationally representative surveys on values and preferences related to independence versus group orientation.

In all models, we also control for having siblings, living with both biological parents and socio-economic status (SES) at age 10. Descriptive statistics for all variables can be found in Appendix D. For the latter, we construct a composite measure of SES in childhood to reflect the multidimensional nature of socio-economic advantage and disadvantage (Cowan et al., 2012). Principal component analysis is run on four indicators measured at age 10: number of rooms (excluding kitchen bathrooms and hallways), number of facilities (fixed bath, cold running water, hot running water, inside toilet and central heating), number of books kept in the home and occupation of the main breadwinner. Parental occupation was collected using the ISCO-08 classification (ILO, 2007), which we use to calculate the Standard International Occupational Prestige Score (SIOPS) (Treiman, 1977). It is a hierarchical scale from 0 to 100, the higher the value, the higher the occupational prestige. This index has the advantage of being stable across cohorts (Lersch et al., 2020), countries (Hout, 2003) and gender (Warren et al., 1998). A similar approach has been used in recent papers using SHARE (Angelini et al., 2019; Havari & Mazzonna, 2015; Mazzonna, 2014). Following the Kaiser Rule, only one factor with an eigenvalue greater than 1 is retained as shown in Appendix E. Our SES index is operationalised into quintile—1 corresponding to the most deprived quintile and 5 the most advantage quintile—to establish whether the association parental SES and loneliness is linear. Because most control variables are measured at age 10, they may have been influenced by migration experiences from birth to age 10. Thus, as a robustnest check, we replicate the analysis for moves between the ages of 10–16 separately in Appendix F.

We use cross-sectional weights for descriptive statistics to obtain results representative of national populations of individuals (Bergmann et al., 2019a, b ). As commonly done in life-course studies (Lynch, 2003; Rosenfeld & Roesler, 2019), our regression analyses remain unweighted because the sampling weights are a function of independent variables (Winship & Radbill, 1994). For ease of interpretation, we report regression coefficients as odds ratios (OR) with 95 per cent confidence intervals. ORs larger (smaller) than one indicate that a given explanatory variable is associated with an increase (decrease) in the likelihood that the outcome is realised compared to the baseline outcome, *ceteris paribus*. For ease of interpretation, results from interaction terms are presented as predicted probabilities, with control variables held at the sample means.

## Empirical Evidence

### Migration History and Loneliness in Childhood

Table 2 reports the results from Model 1. As expected, children who migrated at least once within a NUTS-2 region are 25 per cent more likely to be often lonely and children who changed NUTS-2 region of residence are 39 per cent more likely to be lonely, which lends support to Hypothesis 1a. We note, however, that the regression coefficients are similar for intra and inter-regional migration as indicated by overlapping confidence intervals. This suggests that short- and long-distance migration are equally detrimental to loneliness in childhood, which invalidates Hypothesis 1d.

**Table 2:** Odds ratios, dependent variable often being lonely

	Model 1				Model 3				
	Intra-NUTS-2 migration		Inter-NUTS-2 migration		Intra-NUTS-2 migration		Inter-NUTS-2 migration		
Migration history from birth to 16 years									
Migrated within NUTS-2	1.25***	[1.12,1.38]							
Migrated between NUTS-2			1.39***	[1.21,1.60]					
Duration of residence at address (in years, logged)					0.84***	[0.80,0.89]			
Duration of residence in NUTS-2 (in years, logged)							0.83***		[0.76,0.90]
Demographic characteristics									
Female	1.49***	[1.36,1.64]	1.49***	[1.36,1.64]	1.49***	[1.36,1.63]	1.49***		[1.36,1.64]
Birth cohort (ref. cat. < 1939)									
1939–1945	1.00	[0.87,1.18]	1.02	[0.87,1.19]	1.01	[0.87,1.18]		1.02	[0.87,1.19]
1945 >	1.07	[0.93,1.22]	1.08	[0.95,1.23]	1.06	[0.93,1.21]		1.08	[0.95,1.23]
Both parents are native-born (ref. cat.)									
1 foreign-born parent	0.99	[0.81,1.23]	1.01	[0.82,1.24]	1.00	[0.81,1.22]		1.01	[0.82,1.24]
2 foreign-born parents	0.78	[0.59,1.01]	0.78	[0.59,1.04]	0.78	[0.59,1.03]		0.79	[0.59,1.04]
Socio-economic status at age at 10									
Bottom quintile	1.32**	[1.13,1.54]	1.32**	[1.12,1.54]	1.30**	[1.11,1,52]	1.31**		[1.12,1.53]
Second quintile	1.09	[0.94,1.27]	1.09	[0.94,1.27]	1.09	[0.94,1.26]	1.09		[0.95,1.27]
Third quintile (ref. cat.)	1.00	[1.00,1.00]	1.00	[1.00,1.00]	1.00	[1.00,1.00]	1.00		[1.00,1.00]
Fourth quintile	0.99	[0.86,1.15]	0.99	[0.86,1.14]	0.99	[0.86,1.15]	0.99		[0.86,1.14]
Top quintile	0.98	[0.85,1.13]	0.97	[0.84,1.12]	0.99	[0.85,1.14]	0.98		[0.85,1.13]
Living arrangement									
Siblings	0.65***	[0.58,0.72]	0.64***	[0.57,0.72]	0.64***	[0.58,0.782	0.64***		[0.57,0.72]
Both biological parents	0.83***	[0.78,0.89]	0.83***	[0.77,0.88]	0.83***	[0.78,0.89]	0.82***		[0.77,0.88]
**Country-fixed effect**	X		X		X		X		
Number of observations	37,042								
Log likelihood	− 7,715		− 7,722		− 7,713		− 7,722		

*Source*: Authors’ calculations from Wave 7 of the SHARE. Statistical significance *p < 0.05, **p < 0.01, ***p < 0.001

As a robustness check, we replace our binary measure of loneliness with a four-point scale variable (never lonely, rarely, sometimes and often) and run three regression models for migration within NUTS-2 regions. Results from ordinary least square and ordered logit regression in Appendix A confirm a positive and statistically significant association between moving in childhood and loneliness. Appendix B reports results from multinomial regression using never lonely as the base outcome. It reveals no statistically significant association between moving in childhood and being rarely lonely. The association with migration is marginally statistically significant for being sometime lonely (OR = 1.09, *p* < 0.05) and is clearly statistically significant for being often lonely (OR = 1.25, *p* < 0.001).

Importantly, results for control variables of Model 1 in Table 2 show that living with both biological parents and having siblings exert a strong protective effect on loneliness, more so than socio-economic background. The results are similar when socio-economic background is replaced by parental education (results not reported). Importantly, regression coefficients for family structure are large and similar to that of internal migration: having at least one sibling is associated with a 30 per cent decrease in the odds of often being lonely and living with both biological parents by a 20 per cent decrease. Results from the multinomial regression in Appendix C show that having siblings exerts a protective effect on all levels of loneliness (rarely, sometimes and often) as compared to never being lonely, but its impact is proportionally stronger on being often lonely (OR = 0.59, *p* < 0.001) than rarely lonely (OR = 0.83, *p* < 0.001). The patterns of results are similar for parents. Thus, while internal migration is important, it is only one of multiple factors that collectively shape feelings of loneliness in childhood.

**Fig. 1:**
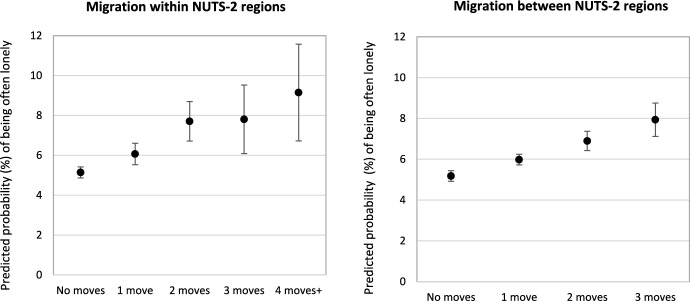
Predicted probability (%) of often being lonely during school years by number of moves and move type with 95% confidence intervals. *Source*: Authors’ calculations from Wave 7 of SHARE. Full regression results can be found in Appendix G. Control variables are held at the sample mean

In Model 2, we replace our binary measure of migration (having migrated or not) by a series of binary variables that capture the number of moves to establish whether the strength of the association increases with the number of past moves. Full result can be found in Appendix G. Predicted probabilities in Fig. 1 shows that, for intra-NUTS-2 migration, the odds of often being lonely increases from 5.14 per cent among children who never moved to 6.07 per cent for those moved once. It plateaus at 7.8 per cent among those who migrated two and three times and increase to 9.15 per cent among those who moved four times or move. For long-distance migration between NUTS-2 regions, the incidence of frequent loneliness increases progressively and plateaus at around 8 per cent among those who migrated three times or more. Thus, the negative association between internal migration and loneliness increases with the number of moves, supporting Hypothesis 1d.

**Fig. 2:**
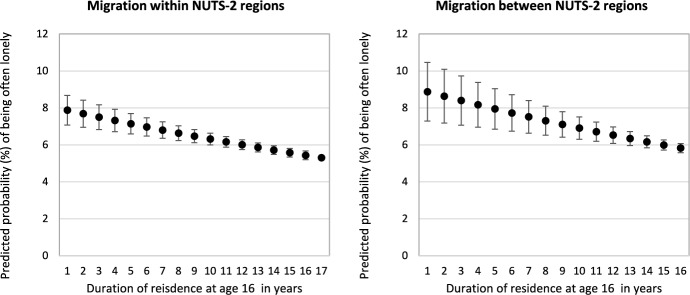
Predicted probability (%) of often being lonely during school years by duration of residence with 95% confidence intervals. *Source*: Authors’ calculations from Wave 7 of SHARE. Regression results can be found in Table 2 (Model 3). Control variables are held at the sample mean

In Model 3, we replace internal migration history by duration of residence. Unsurprisingly, the greater the duration of residence the lower the odds of being often lonely. Results in Table 2 show that the odds ratios are of the same magnitude for duration at the same address and duration in a NUTS-2 region. This indicates that children progressively adjust to their new surroundings, presumably by developing new social networks at destination. This is clearly visible in Fig. 2, which displays predicted probabilities in years of residence as measured at age 16. It shows a clear negative relationship between the probability of often being lonely and duration of residence at both spatial scales. This suggests that the effect of internal migration on loneliness eventually dissipates over time, which validates Hypothesis 1b.

**Fig. 3:**
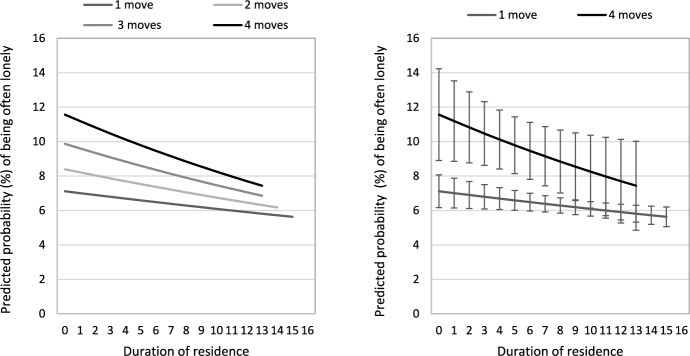
Predicted probability (%) of often being lonely during school years by duration of residence and number of moves within NUTS-2 regions. *Source*: Authors’ calculations from Wave 7 of SHARE. Control variables are held at the sample means

We further test this proposition and analyse the cumulative effect of past moves on loneliness by interacting duration of residence with the number of internal migrations. Figure 3 reports results as predicted probabilities of often being lonely for within NUTS-2 moves because of the limited incidence of repeat childhood long-distance migration. It shows that the probability of often being lonely increases with number of moves, but the association between internal migration and loneliness lessens with duration of residence. However, among children with the same duration of residence, those with higher number of past moves report a higher likelihood of being lonely. For example, among children with a duration of residence of less than a year, 7.12 per cent of those who moved only once often feel lonely compared with 11. 57 per cent of those who moved four times in childhood. The difference in loneliness between one-time migrants and repeat migrants diminishes with duration of residence and converges only after 9 years of residence as shown by overlapping confidence intervals.

In Model 4, we next explore the role of migration timing by distinguishing between pre-school moves before age 6 and moves during school years. Because early movers are more likely to move repeatedly during childhood (Bernard, 2022; Myers, 1999b), we focus on one-time movers and compare them to children who never moved, focussing again on within NUTS-2 moves because of the limited incidence of repeat long-distance migration in childhood. This allows us to disentangle the migration impact of timing from frequency of moves. Results in Appendix H show only moves during school years display a statistically significant association with loneliness (OR = 1.23, *p* < 0.001),^1^ which supports Hypothesis 1c. This aligns with our findings in Fig. 3 showing that the strength of the association between migration and loneliness decreases with duration of residence and research on childhood socialisation that shows that children are effective at building new friendship even in adverse circumstances (Bowker et al., 2010). We conclude that children who move early in life before starting school have ample time to rebuild social networks to the extent that migration is not detrimental to their social networks in the longer term.

### The Moderating Role of Family Structure and Cultural Individualism

**Fig. 4:**
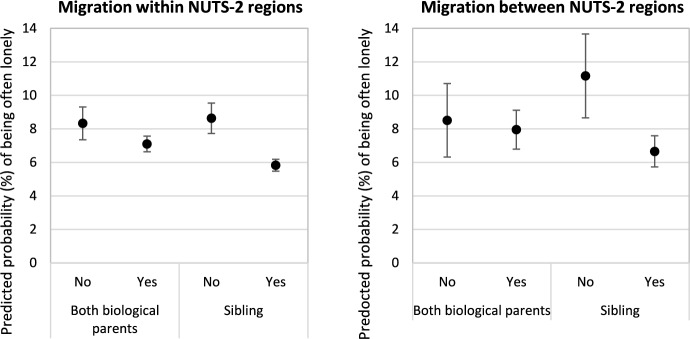
Predicted probability (%) of often being lonely during school years by living arrangements and migration type. *Source*: Authors’ calculations from Wave 7 of SHARE. Control variables are held at the sample means

After having established the association between internal migration and loneliness in childhood and determined variation by migration frequency, timing, duration and distance, we next explore whether the strength of these associations varies depending on family structures and cultural individualism. To that end, we add in Model 5 an interaction term between migration and family structure. Resulting predicted probabilities in Fig. 4 shows that having siblings strongly moderates the negative impact of migration on loneliness, but living with both biological parents does not as shown by overlapping interval, which invalidates Hypothesis 2b. Specifically, children with no sibling who changed NUTS-2 region of residence report a predicted probability of being often lonely of 11.17% compared with a predicted probability of 6.66% among children with siblings, which lends support to Hypothesis 2a.

**Fig. 5:**
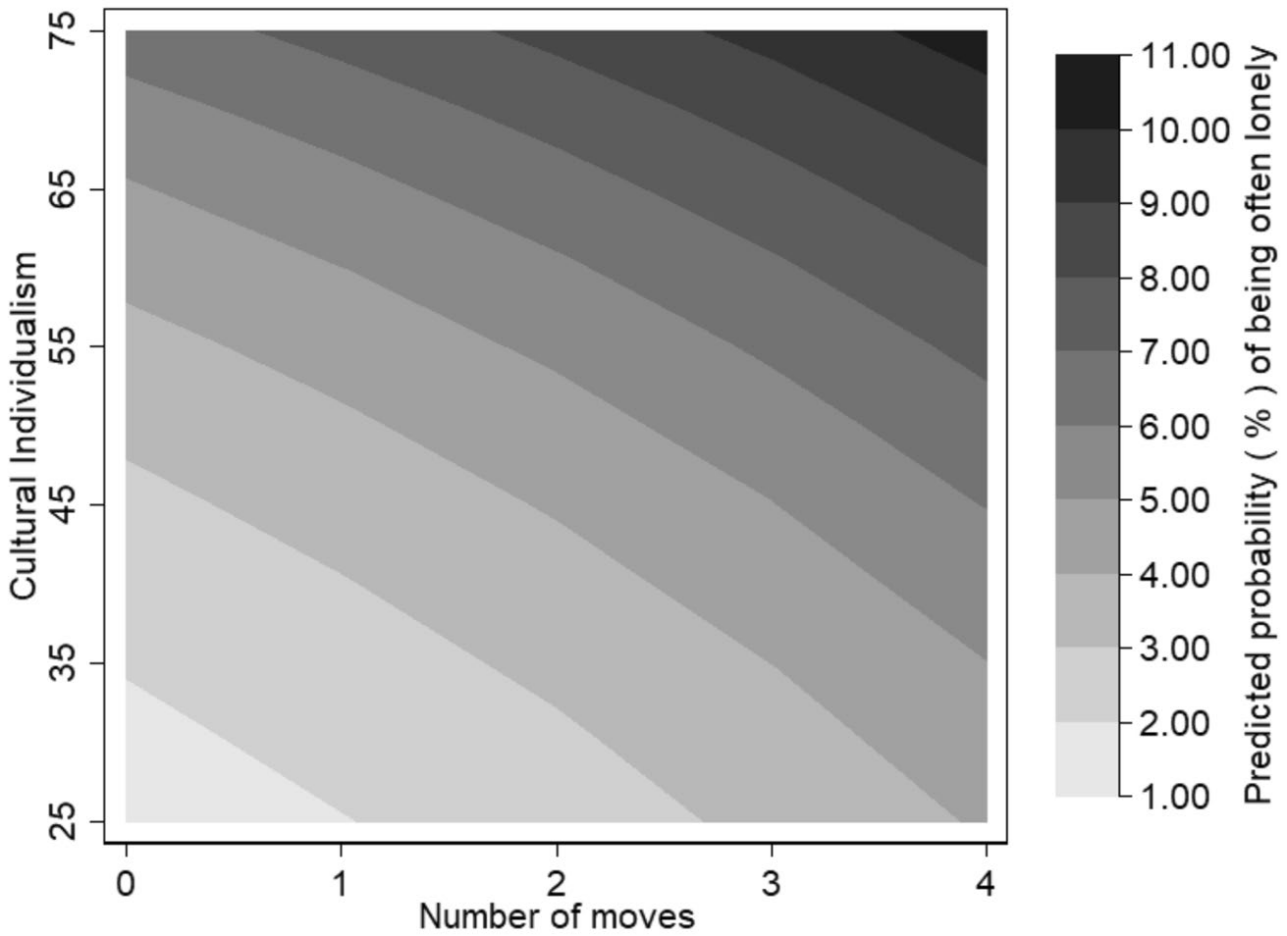
Predicted probability (%) of often being lonely during school years by number of moves within NUTS-2 and level of cultural individualism. *Source*: Authors’ calculations from Wave 7 of SHARE. Control variables are held at the sample means. The lower the cultural individualism score, the stronger a country’s orientation towards collectivist values is. The higher the cultural individualism score, the stronger a country’s orientation towards individualist value is

Finally, we turn our attention to the role of the broader societal context by replacing the country-fixed effect by a country-level measure of cultural individualism, which we interact with migration history in Model 6. Figure 5 displays the predicted probabilities of being often lonely by the number of intra-NUTS-2 moves against the level of cultural individualism, with low scores indicating collectivist values and high scores individualistic values.

First, the results show that the prevalence of childhood loneliness is lower in collectivist societies, which lends support to hypotheses 3a. Among children who never moved those in collectivist countries with an individualism score of 25 such as Portugal and Slovenia have a predicted probability of being often lonely of 1.53% (95% CI 1.24%–1.83%) compared with a predicted probability of 6.50% (95% CI 6.12%–6.88%) for those in countries with an individualism score of 75 such as Belgium and Denmark.

Second, in both collectivist and individualist countries, the prevalence of childhood loneliness increases with the number of moves. For example, in countries with an individualism score of 25, the predicted probability of being often lonely is 4.11% (95% CI 1.44%–6.81%) for children who moved four times compared with 1.53% (95% CI 1.24%–1.83%) for children who never moved. However, the curvature of the contour lines, as opposed to straight lines, show that the association between the number of moves and loneliness differs across levels of cultural individualism. Specifically, the prevalence of loneliness increases proportionally more with the number of moves in collectivistic countries. For example, the incidence loneliness increases by close to a threefold from zero to four moves in countries with an individualism score of 25 (from 1.53 to 4.11%). In contrast, it increases by less than a twofold in countries with an individual score of 75 (from 6.50 to 10.52%). This contradicts Hypothesis 3b which anticipated the association between migration and loneliness to be weaker in collectivist societies because of the greater role of family relationships, which can partially compensate for friendship losses in the short term. A possible explanation for this result is higher social needs in collectivistic societies, which can lead to a mismatch between expected and realised social relationships and results in higher levels of perceived loneliness (Johnson & Mullins, 1987). Conversely, in countries with cultural orientations towards individualism, parents tend to encourage independence in their children (Tamis‐LeMonda et al., 2008), which may result in better coping strategies when moving. Despite the nonlinear association between cultural values, migration and loneliness, we observe that, overall, children living in collectivist societies are comparatively less affected by migration, even if they move multiple times because of the lower baseline level of loneliness.

## Discussion and Conclusion

Building on a growing literature on the consequences of childhood migration on life outcomes, we have explored an under-researched consequence of internal migration, loneliness. This has served to test the loss-of-social-networks theory, which is commonly used to explain the negative association between childhood migration and life outcomes. Compared with previous work, our study also has the unique advantage of using a cross-national dataset to tease out the role of culture in moderating the association between internal migration and loneliness in order to provide a more comprehensive understanding of childhood loneliness and to pinpoint the circumstances under which children are more greatly affected by internal migration. Results are listed in summary form against hypotheses in Table 3.

**Table 3:** Hypotheses on the links between internal migration and loneliness in childhood

Hypothesis		Validation	Findings
1a	Internal migration is associated with higher levels of loneliness.	Yes	Children who migrated at least once within a NUTS-2 region are 25 per cent more likely to be often lonely and children who changed NUTS-2 region of residence are 39 per cent more likely to be often lonely.
1b	The association between migration and loneliness decreases with duration of residence.	Yes	The greater the duration of residence the lower the odds of being often lonely. This is true for both intra-NUTS-2 and inter-NUTS-2 migration.
1c	Migration during school years is more strongly associated with loneliness than pre-school migration.	Yes	The association between migration and loneliness is statistically significant only for moves after age 6.
1d	The association between migration and loneliness increases with the number of moves.	Yes	This pattern of results holds for both intra-NUTS-2 and inter-NUTS-2 migration. However, the strength of the association between the number of moves and loneliness diminishes with duration of residence.
1e	Long-distance moves are more strongly associated with loneliness than short-distance relocations.	No	The difference between intra- and inter-NUTS-2 moves is not statistically significant.
2a	The association between migration and loneliness is lower in multi-child families than in single-child families.	Yes	Children with siblings are less likely to be lonely and the presence of sibling reduces the strength of the association between internal migration and loneliness. Children with no sibling who changed NUTS-2 region have a predicted probability of being often lonely of 11.17% compared with a predicted probability of 6.66% for children with siblings.
2b	The association between migration and loneliness is lower in two-parent families than in single-parent families.	No	Children who live with both parents are less likely to be often lonely, but living with both parents does not reduce the strength of the association between migration and loneliness.
3a	The prevalence of childhood loneliness is higher in countries with cultural orientations towards individualism.	Yes	Among children who never moved, the predicted probability of being often lonely is under 3% for those in countries with cultural orientation towards collectivism such as Portugal and Slovenia but is close to 6% in individualist countries like Belgium and Denmark.
3b	The association between migration and loneliness is weaker in collectivist countries.	Mixed	In both collectivist and individualist countries, the prevalence of childhood loneliness increases with the number of moves. Collectivist countries display lower levels of loneliness for all number of moves. However, the association between the number of moves and loneliness increases comparatively more in collectivist countries.

Our findings reveal that children who have experienced internal migration, whether short or long-distance, face a higher likelihood of experiencing loneliness during their school years. We have found that while living with both biological parents and having siblings act as protective factors by reducing reported loneliness, only having siblings reduces the negative association of internal migration on loneliness. These results are for cohorts born before 1967, but they have important implications for our contemporary understanding of migration-induced loneliness. Given the long-term decline in fertility fuelled by falling first and second-birth rates in Europe (Zeman et al., 2018), we expect—everything else being equal—the share of lone children to be on the rise and consequently the impact of internal migration on loneliness to have increased at the population level considering that a growing number of children do not benefit from sibling support.

Our results also suggest that longer periods of residence mitigate the loneliness associated with migration. However, when considering the cumulative impact of multiple relocations, those with frequent moves are at a heightened risk even after controlling for duration of residence. These findings align with prior explanations for why moving can be challenging for children, including theories on social-network-loss. Frequent moves can disrupt peer relationships and family networks, hinder the formation of lasting friendships, and foster feelings of isolation. Conversely, those who move just once tend to have a lower probability of experiencing loneliness, possibly because of a more stable social environment. A longer duration of residence gives children more time to adapt to their new surroundings and establish new friendships. Nevertheless, the lasting effects of multiple moves remain evident. Our result also show that the timing of internal migration matters as moves before age 6 are not associated with higher levels of loneliness in school years. This finding aligns with research on increased risk of moving in late childhood for educational attainment and mental health (Fowler et al., 2015; Myers, 1999a; Rumbold et al., 2012).

As outlined in the Introduction, childhood relocations have been associated with adverse consequences both in adolescence and in young adulthood, including behavioural issues (Böhlmark, 2009), school drop-out, (McMullin et al., 2021; Tonnessen et al., 2016), early parenthood, reduced adult income (Tonnessen et al., 2016), and variable health repercussions (Tseliou et al. 2021). Equally important, childhood loneliness has been found to be a precursor to higher level of loneliness among young adults (Oishi et al., 2012). By providing valuable insights into the association between childhood residential mobility and feelings of loneliness, this study contributes to a better understanding of the mechanisms of the adverse effects of moving on a child’s future prospects, while highlighting the social risks associated with repeat migration in childhood.

By considering cultural values, we have also shown that the prevalence of childhood loneliness is higher in countries with cultural orientations towards individualism, which value self-reliance and encourage loose social networks dominated by chosen relationships, as opposed to collectivist cultures which support tighter social networks dominated by family members. This demonstrates the importance of cross-national perspectives and the need to control for variations in the baseline level of loneliness when analysing the association between internal migration and loneliness. Importantly, we have found that collectivist countries consistently display lower levels of loneliness than individualist countries, whether children never moved or moved multiple times. However, the association between the number of moves and loneliness increases comparatively more in collectivist countries. This contradicts our theoretical expectation that the greater role of family relationships in collectivist cultures would partially compensate for migration-related friendship losses. Instead, our results suggest that higher social needs in collectivistic societies may lead to a mismatch between expected and realised social relationships (Johnson & Mullins, 1987) and result in higher levels of perceived loneliness among repeat movers. Conversely, in countries with cultural orientations towards individualism, parents tend to encourage independence in their children (Tamis‐LeMonda et al., 2008). This may result in better coping strategies when moving and explain why repeat child movers are proportionally less affected by migration, but yet report the highest absolute level of loneliness.

By jointly considering how the association between internal migrations varies by migration frequency, timing, duration and distance and unpacking the moderating role of family structures and cultural individualism, we can identify the children at higher risk of migration-induced loneliness. These are children with no sibling who reside in countries with cultural orientations towards individualism and in countries with high level of internal migration, where more children move and do so multiple times (Bell, 1996; Bernard, 2017; Goldstein, 1954, 1964; Morrison, 1971; Pertzikovitz et al., 2024). The rise in single-child families coupled with a global rise in individualism (Santos et al., 2017)—even if countries’ scores relative to one another have been stable over time (Beugelsdijk et al., 2015)—means that the impact of internal migration on loneliness is likely to be greater for children today than for the birth cohorts studied in this paper. Importantly, highly mobile countries typically score high on individualism (Buttrick & Oishi, 2021). This is the case in Europe where the spatial gradient of high mobility in the north and west moderating to low mobility in the south and east (Bell et al., 2015; Sánchez & Andrews, 2011) mirrors spatial variations in individualism (Hofstede, 2001). Thus, children in single families in countries like France, Belgium, Denmark and Sweden are likely to be the most affected by loneliness-migration.

The decline in the level of internal migration observed over the last 40 years particularly in Anglo-Saxon countries (Champion et al., 2018; Cooke, 2013; Foster, 2017; Kalemba et al., 2020) and to a lesser extend in Northern Europe (Garðarsdóttir et al., 2020; Shuttleworth et al., 2017) may have limited the impact of fertility and cultural trends on migration-induced loneliness. However, the decline in internal migration is not universal (Bell et al., 2018) and some European countries have recorded stable or increasing levels of internal migration (Alvarez et al., 2021). Considering how these macro trends will combine to shape future migration-induced loneliness warrants further research. This is particularly important given that we focussed on pre-1967 cohorts whose childhood preceded the emergence of digital technologies. These may help limit the severance of friendships caused by migration and in turn moderate the social consequences of moving. While digital tools may help maintain long-distance relationships, they can also hinder local social integration. Children who rely heavily on online communication may struggle to build strong offline networks in their new environment. However, research on digital technology and friendship is a relatively recent topic of inquiry and the implications of digital technology in the context of migration are yet to be fully considered.

Despite its contributions, our paper faces several limitations. The first one is the reliance on retrospective data, which means that our results are conditional on survival to the date of the survey. Because loneliness in childhood is a precursor to mental health issues later in life, it is possible that some individuals may have died before the interview, which may bias the association between migration and loneliness downward. While SHARE retrospectively collected information from Europeans aged 50 and older using life-history calendar grids to aid recall, we cannot completely rule out recall bias even if migration is a salient event. Additionally, while it might be difficult for respondents to pinpoint the exact timing of their feeling of loneliness, it is arguably easier to recall more intense feelings that last over an extensive period. Because our retrospective measure of childhood loneliness may be affected by post hoc rationalisation, we replicated the analysis for two sub-groups: those who reported being often lonely in adulthood at the time of the survey and those who did not. Results in Appendix C show that the association between loneliness and internal migration is statistically significant for both groups, which indicates that our results are not significantly affected by the retrospective nature of childhood loneliness.

Secondly, the study controls for parental circumstances only at age 10, making it difficult to exactly pinpoint the mechanisms through which internal migration influences loneliness. To partially address this, we conducted a robustness check between the ages of 10–16 and controlled for family situation and living arrangements. Results in Appendix F confirm the negative association between internal migration and childhood migration. However, we cannot fully capture all family changes between the ages 10 and 16, resulting in the association between the number of migrations, duration of residence and loneliness being perhaps slightly over-estimated. This is compounded by the fact that loneliness is not measured at a specific point in time in SHARE, which further limits our ability to claim any causal link between migration and loneliness. Linked to this is the risk of reserved causality that may arise if friendship and mental health issues prompt families to move elsewhere, which could not be investigated here. Future longitudinal prospective surveys of children, including those enriched with administrative data in Scandinavia and the Netherlands or datasets such as CILS4EU^2^ can potentially provide an opportunity to tease out these issues in future research and may also be used to explore the role of extended family members in mitigating migration-induced loneliness, which could not be done here. Such datasets would also have the advantage of providing more detailed geospatial information, which should permit a more fine-grained measurement of short- and long-distance migration. Indeed, our definition of short-distance migration as a change of address within NUTS-2 may still be capturing some longer-distance moves given the size of some NUTS-2 regions.

Despite these limitations, this study offers unique insights into the cumulative effect of past moves. Our findings underscore the potential role of residence duration in mitigating the influence of past moves on feelings of loneliness, which can significantly affect overall mental health and other associated outcomes. Furthermore, prior research on the intergenerational transmission of migration behaviours has established that experiencing migration at least once in childhood is sufficient to increase the odds of migrating in adulthood (Bernard & Vidal, 2020) potentially supporting the development of skills promoting positive experiences of migration for education and employment in later life.

Overall, our results support the loss-of-social networks hypothesis, suggesting that children who move less frequently and have time to adapt to their new surroundings are less prone to loneliness. Those with siblings are better shielded from the negative effects of moving on feelings of loneliness, whereas those who experience multiple moves are more vulnerable. In summary, a single childhood migration may have limited effects on loneliness and can even foster valuable skills by learning how to leave and enter new social contexts. Conversely, those who move often during childhood are at a higher risk of more adverse consequences. Thus, it is important to identify and support those children most at risk of isolation in their new location, particularly in countries with a cultural orientation towards individualism.

## Appendix A: Regression coefficients of loneliness during school years measured on a four-point scale from OLS regression and ordered logit regression with 95% confidence intervals

**Table:**

	OLS		Ordered logit	
Moved at least once within NUTS-2 from birth to age 16	0.05***	[0.03,0.07]	0.08**	[0.03,0.13]
Demographic characteristics				
Female	0.11***	[0.09,0.12]	0.21***	[0.17,0.25]
Birth cohort (ref. cat. < 1939)				
1939–1945	− 0.01	[− 0.04,0.02]	− 0.02	[− 0.10,0.05]
1945 >	0.02*	[0.00,0.06]	0.07*	[0.01,0.13]
Both parents are native-born (ref. cat.)				
1 foreign-born parent	− 0.02	[− 0.07,0.02]	− 0.05	[− 0.15,0.05]
2 foreign-born parents	− 0.15***	[− 0.20,-0.10]	-0.37***	[− 0.48,-0.25]
Socio-economic status at age at 10				
Bottom quintile	0.06***	[0.03,0.09]	0.10**	[0.04,0.17]
Second quintile	0.00	[− 0.02,0.04]	0.02	[− 0.05,0.08]
Third quintile (ref. cat.)				
Fourth quintile	− 0.00	[− 0.03,0.03]	− 0.01	[0.08,0.05]
Top quintile	− 0.03*	[− 0.06,-0.00]	-0.07*	[− 0.14,-0.01]
Living arrangement				
Siblings	− 0.16***	[− 0.19,-0.14]	− 0.34***	[− 0.39,-0.28]
Both biological parents	− 0.04***		− 0.08***	[− 0.11,-0.04]
Constant	4.81***	[4.75,4.85]		
Constant (cut 1)			0.06	[− 0.08,0.20]
Constant (cut 2)			1.23***	[1.00,1.27]
Constant (cut 3)			2.58***	[2.43.2.72]
Country-fixed effect	Yes		Yes	
Number of observations	37,052		37,052	
Log likelihood	− 48,757		− 39,361	

*Source*: Authors’ calculations from Wave 7 of the SHARE, regression coefficients are not exponentiated. Four-point scale measure of loneliness: often, sometimes, rarely and never.

## Appendix B: Odds ratio of loneliness during school years measured on a four-point scale from multinomial regression, with as never lonely as the base outcome and 95% confidence intervals

**Table:**

	Rarely lonely		Sometimes lonely		Often lonely	
Moved at least once within NUTS-2 from birth to age 16	0.97	[0.90,1.03]	1.09 *	[1.01,1.17]	1.25***	[1.13,1.39]
Demographic characteristics						
Female	1.06*	[1.01,1.12]	1.28***	[1.20,1.36]	1.58***	[1.43,1.73]
Birth cohort (ref. cat. < 1939)						
1939–1945	0.97	[0.88,1.06]	0.98	[0.87,1.09]	0.99	[0.85,1.16]
1945 >	1.02	[0.94,1.07]	1.12	[1.02,1.22]	1.08	[0.95,1.24]
Both parents are native-born (ref. cat.)						
1 foreign-born parent	0.94	[0.63,0.85]	0.91	[0.79,1.07]	0.96	[0.78,1.18]
2 foreign-born parents	0.73***	[0.90,1.07]	0.63***	[1.03,1.26]	0.67***	[0.51,0.90]
Socio-economic status at age at 10						
Bottom quintile	0.98	[0.90,1.07]	1.14*	[1.03,1.26]	1.34***	[1.14,1.56]
Second quintile	0.97	[0.90,1.06]	1.02	[0.93,1.13]	1.10	[0.94,1.27]
Third quintile (ref. cat.)						
Fourth quintile	0.99	[0.91,1.08]	0.99	[0.92,1.09]	0.98	[0.85,1.14]
Top quintile	0.92*	[0.84,0.99]	0.93	[0.84,1.02]	0.94	[0.81,1.09]
Living arrangement						
Siblings	0.83***	[0.77,0.89]	0.68***	[0.62,0.73]	0.58***	[0.52,0.65]
Both biological parents	1.01	[0.96,1.05]	0.95*	[0.90,0.99]	0.83***	[0.77,0.88]
Country-fixed effect	Yes					
Number of observations	37,042					
Log likelihood	− 39,073					

*Source*: Authors’ calculations from Wave 7 of the SHARE. Four-point scale measure of loneliness: often, sometimes, rarely and never.

Statistical significance * *p* < 0.05, ** *p* < 0.01, *** *p* < 0.001.

## Appendix C: Odds ratios of being often lonely during school years with 95% confidence interval by level of loneliness at the time of the survey

**Table:**

	Intra-NUTS-2 migration					Inter-NUTS-2 migration			
	Often lonely at the time of the survey			Not often lonely at the time of the survey		Often lonely at the time of the survey		Not often lonely at the time of the survey	
Migration history birth to age 16	1.25*	[1.03,1.52]		1.19***	[1.12,1.28]	1.73*	[1.07,2.80]	1.35***	[1.17,1.57]
Demographic characteristics									
Female	1.25	[0.87,1.80]		1.79***	[1.33,1.62]	1.25	[0.87,1.80]	1.48***	[1.33,1.62]
Birth cohort (ref. cat. < 1939)									
1939–1945	0.97	[0.55,1.69]		1.03	[0.88,1.22]	0.98	[0.56,1.71]	1.03	[0.88,1.22]
1945 >	2.03**	[1.31,3.14]		1.04	[0.90,1.19]	2.06**	[1.33,3.19]	1.05	[0.91,1.20]
Both parents are native-born (ref. cat.)									
1 foreign-born parent	1.26	[0.67,2.48]		0.97	[0.78,1.20]	126	[0.64,2.49]	0.99	[0.79,1.22]
2 foreign-born parents	0.76	[0.31,1.88]		0.76	[0.57,1.03]	0.77	[0.30,1.89]	0.77	[0.57,1.03]
Socio-economic status at age at 10									
Bottom quintile	1.16	[0.67,2.00]		1.31***	[1.12,1.54]	1.12	[0.68,1.93]	1.31***	[1.12,1.54]
Second quintile	1.10	[0.66,1.85]		1.10	[0.94,1.28]	1.13	[0.97,1.88]	1.10	[0.94,1.28]
Third quintile (ref. cat.)	1.00	[1.00,1.00]		1.00	[1.00,1.00]	1.00	[1.00,1.00]	1.00	[1.00,1.00]
Fourth quintile	0.99	[0.59,1.67]		1.01	[0.87,1.17]	0.98	[0.58,1.64]	1.00	[0.86,1.17]
Top quintile	0.89	[0.53,1.49]		1.00	[0.89,1.17]	0.87	[0.52,1.43]	0.99	[0.85,1.16]
Living arrangement									
Siblings	0.76***	[0.50,1.14]		0.64***	[0.57,0.72]	0.74	[0.49,1.11]	0.64***	[0.57,0.72]
Both biological parents	0.73***	[0.59,0.91]		0.52***	[0.79,0.891]	0.73***	[0.58,0.91]	0.84***	[0.78,0.91]
Country-fixed effect	YES			YES	YES	YES	YES	YES	
Number of observations	1,273		35,765			1,273		35,765	
Log likelihood	− 494		− 7142			− 494		− 7,142	

*Source*: Authors’ calculations from Wave 7 of the SHARE, statistical significance * *p* < 0.05, ** *p* < 0.01, *** *p* < 0.001.

## Appendix D: Descriptive statistics

**Table:**

	%	Mean	Standard deviation
Loneliness during school years			
Often	5.66		
Sometimes	14.36		
Rarely	21.30		
Never	58.68		
Migration history from birth to age 16			
Migration within NUTS-2			
0 move	76.05		
1 move	16.92		
2 moves	5.00		
3 moves	1.35		
4 moves +	0.67		
Moved at least once before age 6	9.25		
Moved at least once between age 6 and 16	18.67		
Mean duration of residence at age 16 (in years logged)	–	2.47	0.74
Migration between NUTS-2			
0 move	90.79		
1 move	7.40		
2 moves	1.42		
3 moves	0.39		
4 moves +	–		
Moved at least once before age 6	3.36		
Moved at least once between age 6 and 16	6.75		
Mean duration of residence at age 16	–	2.73	0.44
Demographic characteristics			
Female	56.05		
Male	43.95		
Birth cohort			
< 1939	15.09		
1939–1945	17.37		
1945 >	67.54		
Immigrant status			
Both parents are native-born	91.27		
1 foreign-born parent	4.56		
2 foreign-born parents	4.17		
Socio-economic status at age at 10			
Bottom quintile	19.70		
Second quintile	19.87		
Third quintile (ref. cat.)	19.93		
Fourth quintile	20.15		
Top quintile	20.36		
Living arrangement at age 10			
Lived with siblings	83.84		
Lived with both biological parents	89.68		
Cultural individualism		56.89	15.13

*Source*: Authors’ calculations from Wave 7 of the SHARE, unweighted data.

## Appendix E Factor loading for the construction of an index of socio-economic status at age 10

**Table:**

Variables	Factor loading
Number of rooms excluding kitchen and bathrooms	0.65
Number of books	0.76
Number of facilities (fixed bath, cold running water, water supply, hot running water supply, inside toilet and central heating)	0.78
Breadwinner occupational prestige	0.55

Principal component analysis on Wave 7 data from SHARE using an orthogonal rotation. Factors with an eigenvalues greater than one were retained.

## Appendix F: Odds ratios of being often lonely during school years with 95% confidence intervals, controlling for migration between ages 10 to 16

**Table:**

	**Intra-NUTS-2 migration**	
Moved between ages 10–16	1.36***	[1.21,1.53]
Demographic characteristics		
Female	1.49***	[1.36,1.63]
Birth cohort (ref. cat. < 1939)		
1939–1945	1.01	[0.87,1.18]
1945 >	1.06	[0.93,1.21]
Both parents are native-born (ref. cat.)		
1 foreign-born parent	1.00	[0.81,1.22]
2 foreign-born parents	0.78	[0.59,1.03]
Socio-economic status at age at 10		
Bottom quintile	1.31***	[1.12,1.52]
Second quintile	1.09	[0.94,1.27]
Third quintile (ref. cat.)		
Fourth quintile	0.99	[0.86,1.15]
Top quintile	0.99	[0.86,1.15]
Living arrangement		
Siblings	0.65***	[0.58,0.72]
Both biological parents	0.83***	[0.78,0.88]
Country-fixed effect	Yes	
Number of observations	37,072	
Log likelihood	− 7,718	

*Source*: Authors’ calculations from Wave 7 of the SHARE, analysis restricted to individuals who never moved in childhood or moved only once. Individuals who removed multiple times were removed from the analysis. Statistical significance * *p* < 0.05, ** *p* < 0.01, *** *p* < 0.001.

## Appendix G: Odds ratios of being often lonely during school years with 95% confidence intervals controlling for the number of childhood moves

**Table:**

	Intra-NUTS-2 migration		Inter-NUTS-2 migration	
Migration history (ref. cat. no move)				
1 move	1.20**	[1.07,1.34]	1.27**	[1.08,1.49]
2 moves	1.55**	[1.33,1.81]	1.90***	[1.42,2.54]
3 moves	1.58***	[1.22,2.03]	1.78*	[1.03,3.09]
4 moves +	1.88	[1.38,2.56]		
Demographic characteristics				
Female	1.49***	[1.36,1.64]	1.49***	[1.36,1.64]
Birth cohort (ref. cat. < 1939)				
1939–1945	1.01	[0.86,1.18]	1.01	[0.87,1.18]
1945 >	1.06	[0.93,1.21]	1.08	[0.95,1.23]
Both parents are native-born (ref. cat.)				
1 foreign-born parent	0.98	[0.80,1.21]	1.05	[0.82,1.23]
2 foreign-born parents	0.77	[0.5831.03]	0.78	[0.59,1.04]
Socio-economic status at age at 10				
Bottom quintile	1.31***	[1.13,1.53]	1.32***	[1.13,1.54]
Second quintile	1.10	[0.95,1.27]	1.098	[0.95,1.27]
Third quintile (ref. cat.)				
Fourth quintile	0.99	[0.85,1.14]	0.99	[0.85,1.14]
Top quintile	0.97	[0.84,1.12]	0.97	[0.85,1.12]
Living arrangement				
Siblings	0.65***	[0.582,0.73]	0.64***	[0.58,0.72]
Both biological parents	0.84***	[0.79,0.90]	0.83***	[0.78,0.89]
Country-fixed effect	Yes		Yes	
Number of observations	37,052		37,052	
Log likelihood	− 7,706		− 7,717	

*Source*: Authors’ calculations from Wave 7 of the SHARE.

Statistical significance * *p* < 0.05, ** *p* < 0.01, *** *p* < 0.001

## Appendix H: Odds ratios of being often lonely during school years with 95% confidence intervals, controlling for migration timing

**Table:**

	Intra-NUTS-2 migration	
Intra-NUTS-2 migration (ref. cat. No moves)		
Moved at least once before age 6	0.99	[0.80,1.22]
Moved at least once between age 6 and 16	1.23***	[1.09,1.39]
Demographic characteristics		
Female	1.46***	[1.32,1.62]
Birth cohort (ref. cat. < 1939)		
1939–1945	1.01	[0.85,1.20]
1945 >	1.05	[0.91,1.20]
Both parents are native-born (ref. cat.)		
1 foreign-born parent	0.95	[0.75,1.20]
2 foreign-born parents	0.67*	[0.48,0.92]
Socio-economic status at age at 10		
Bottom quintile	1.27**	[1.08,1.50]
Second quintile	1.08	[0.92,1.26]
Third quintile (ref. cat.)		
Fourth quintile	0.980	[0.84,1.18]
Top quintile	1.00	[0.85,1.18]
Living arrangement		
Siblings	0.64***	[0.57,0.72]
Both biological parents	0.84***	[0.78,0.91]
Country-fixed effect	Yes	
Number of observations	33,277	
Log likelihood	− 6,589	

*Source*: Authors’ calculations from Wave 7 of the SHARE, analysis restricted to individuals who never moved in childhood or moved only once. Individuals who removed multiple times were removed from the analysis. Statistical significance * *p* < 0.05, ** *p* < 0.01, *** *p* < 0.001.

## Footnotes

1 Because most control variables are measured at age 10, they may have been influenced by migration experiences from birth to age 10. Thus, as a robustnest check, we replicate the analysis for moves between the ages of 10–16. Results in AppendiX G (OR = 1.36, *p* < 0.001)confirm the positive and statistically significant association between internal migration and loneliness in chilhood.

2 Henkens et al. (2024) uses German data from wave 6 and wave 8 of Children of Immigrant Longitudinal Study (CILS4EU-DE v.6.0.0) to construct retrospective mobility histories and examine life satisfaction in emerging adulthood.
